# Hippocampal CA3 Transcriptome Signature Correlates with Initial Precipitating Injury in Refractory Mesial Temporal Lobe Epilepsy

**DOI:** 10.1371/journal.pone.0026268

**Published:** 2011-10-14

**Authors:** Silvia Y. Bando, Maryana C. Alegro, Edson Amaro, Alexandre V. Silva, Luiz H. M. Castro, Hung-Tzu Wen, Leandro de A. Lima, Helena Brentani, Carlos Alberto Moreira-Filho

**Affiliations:** 1 Department of Pediatrics, Faculdade de Medicina da Universidade de São Paulo (FMUSP), São Paulo, São Paulo, Brazil; 2 Laboratory of Integrated Systems, Escola Politécnica da Universidade de São Paulo, São Paulo, São Paulo, Brazil; 3 Department of Radiology, Faculdade de Medicina da Universidade de São Paulo (FMUSP), São Paulo, São Paulo, Brazil; 4 Department of Biosciences, Universidade Federal de São Paulo, Santos, São Paulo, Brazil; 5 Clinical Neurology Division, Hospital das Clínicas da Faculdade de Medicina da Universidade de São Paulo, São Paulo, São Paulo, Brazil; 6 Epilepsy Surgery Group, Hospital das Clínicas da Faculdade de Medicina da Universidade de São Paulo, São Paulo, São Paulo, Brazil; 7 Laboratory of Biotechnology, Hospital do Câncer AC Camargo, São Paulo, São Paulo, Brazil; 8 Department of Psychiatry, Instituto Nacional de Psiquiatria do Desenvolvimento and Laboratório de Investigação Médica 23, Faculdade de Medicina da Universidade de São Paulo (FMUSP), São Paulo, São Paulo, Brazil; Massachusetts General Hospital and Harvard Medical School, United States of America

## Abstract

**Background:**

Prolonged febrile seizures constitute an initial precipitating injury (IPI) commonly associated with refractory mesial temporal lobe epilepsy (RMTLE). In order to investigate IPI influence on the transcriptional phenotype underlying RMTLE we comparatively analyzed the transcriptomic signatures of CA3 explants surgically obtained from RMTLE patients with (FS) or without (NFS) febrile seizure history. Texture analyses on MRI images of dentate gyrus were conducted in a subset of surgically removed sclerotic hippocampi for identifying IPI-associated histo-radiological alterations.

**Methodology/Principal Findings:**

DNA microarray analysis revealed that CA3 global gene expression differed significantly between FS and NFS subgroups. An integrative functional genomics methodology was used for characterizing the relations between GO biological processes themes and constructing transcriptional interaction networks defining the FS and NFS transcriptomic signatures and its major gene-gene links (hubs). Co-expression network analysis showed that: i) CA3 transcriptomic profiles differ according to the IPI; ii) FS distinctive hubs are mostly linked to glutamatergic signalization while NFS hubs predominantly involve GABAergic pathways and neurotransmission modulation. Both networks have relevant hubs related to nervous system development, what is consistent with cell genesis activity in the hippocampus of RMTLE patients. Moreover, two candidate genes for therapeutic targeting came out from this analysis: SSTR1, a relevant common hub in febrile and afebrile transcriptomes, and CHRM3, due to its putative role in epilepsy susceptibility development. MRI texture analysis allowed an overall accuracy of 90% for pixels correctly classified as belonging to FS or NFS groups. Histological examination revealed that granule cell loss was significantly higher in FS hippocampi.

**Conclusions/Significance:**

CA3 transcriptional signatures and dentate gyrus morphology fairly correlate with IPI in RMTLE, indicating that FS-RMTLE represents a distinct phenotype. These findings may shed light on the molecular mechanisms underlying refractory epilepsy phenotypes and contribute to the discovery of novel specific drug targets for therapeutic interventions.

## Introduction

Epilepsy affects 50 million people worldwide, 80% of them living in developing countries, and about 30% of the patients did not achieve remission with the available anti-epileptic drugs [1]. Mesial temporal lobe epilepsy (MTLE) is the most common partial epilepsy in adults and hippocampal sclerosis (HS) constitutes its most frequent pathological abnormality [2]. MTLE is often resistant to antiepileptic drugs (refractory epilepsy) and is associated with a history of prolonged febrile seizures (PFS) in childhood or other initial precipitating injuries [2], [3]–[5].

A large body of experimental data on how antecedent febrile seizures lead to MTLE emerged from studies on animal models of inflammation- and hyperthermia-induced seizures [6]–[10]. Some of these studies combined in vitro and animal models in order to check for pre-existing factors, such as ion channel mutations, cortical dysplasia or brain injuries. These factors may explain some but not the vast majority of MTLE cases in which children without any identifiable predisposing factor had PFS and further developed epilepsy. Experimental investigations also focused seizure duration, showing that in animal models PFS determines an increased risk for developing MTLE [8], [9]. This has been confirmed by clinical research, thus indicating that aborting febrile seizures to prevent status epilepticus is an important preventive approach [11]. Subsequently, experimental evidences rendered clear that MTLE arises after febrile seizures through a succession of events, probably starting with the release of inflammatory mediators, what is later followed by cell loss at the hippocampus, mesial temporal sclerosis and neuronal circuitry remodeling [10], [12], [13].

Therefore, the molecular pathomechanisms involved in PFS-induced MTLE would be provoked by inflammatory mediators and then driven by coordinate changes in the expression of hundreds of genes in the brain. With the aim of characterizing these transcriptional changes, DNA microarray methodologies were employed in a variety of animal model studies [7], together with genetic linkage studies conducted in familial PFS-cases [14]. However, for obtaining predictive biomarkers of epileptogenesis and identifying novel therapeutic targets it is mandatory to conduct gene expression studies on surgical specimens obtained from the hippocampi of patients submitted to epilepsy surgery [5], [15].

Patients with refractory MTLE (RMTLE) generally undergo epilepsy surgery and brain explants obtained through this procedure are a valuable material for investigating the molecular mechanisms underlying refractory epilepsy. Studies in surgical specimens helped to reveal the pivotal role of the hippocampus for temporal lobe epilepsies and, particularly, of the dentate gyrus in several biological processes related to the startup and to the end stages of the disease [16]. Pathomorphological studies showed compromised neurogenesis and significant dentate granule cell loss in children and adult patients presenting temporal lobe epilepsy [17]–[19].

**Figure 1:**
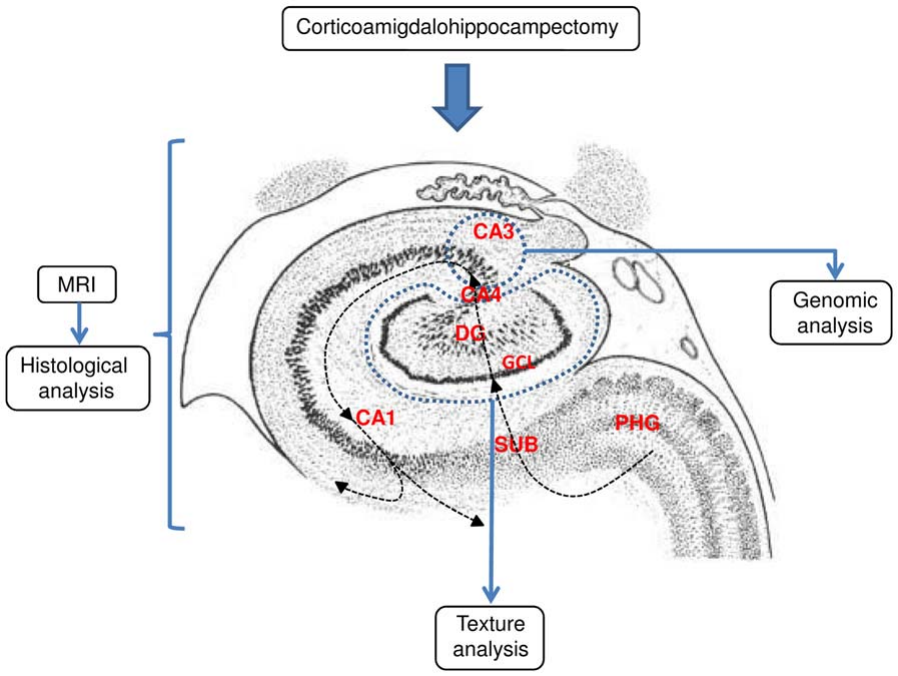
Study work-flow. During epilepsy surgery tissue explants from CA3 were obtained for genomic studies. The entire hippocampus was then resected and the specimen was submitted to MRI for texture analysis of the dented gyrus (DG) and, subsequently, to global histological analysis. Arrowed dotted lines indicate PHG-CA1 input-output trajectory. The blue dotted line indicates CA3 and DG limits. Abbreviations: SUB subiculum; GCL, granule cell layer.

In order to gain a better understanding on the biological and molecular mechanisms governing RMTLE, several authors conducted gene expression studies on surgical specimens obtained from the hippocampus and entorhinal cortex of patients submitted to epilepsy surgery [20]–[23]. These studies, recently reviewed by Wang et al. [5], brought an impressive amount of information but also showed low consistence of data among different laboratories. In order to overcome this flaw those authors suggested that genomic profile studies should be centered in specific anatomic subregions of the hippocampus. Moreover, due to the importance of the initial precipitating injury (IPI) in MTLE development, a comparative analysis of cases with and without prolonged febrile seizure history would be very relevant. Here we pursued this kind of analysis by studying the transcriptomic profile of hippocampal CA3–CA4 transition explants obtained surgically from patients with RMTLE and HS with (FS) or without (NFS) a history of febrile seizures as the IPI. High-resolution 3T magnetic resonance imaging (MRI) texture analysis was employed in order to assess IPI-related differences in the surgical removed hippocampi of FS and NFS patients. A summary of the study work-flow is depicted in Figure 1.

## Results

### Patients' clinical and pathological data

A total of 23 patients with refractory epilepsy and mesial temporal sclerosis who underwent corticoamygdalohippocampectomy were included in this study. Their main clinical features are summarized in Table 1. None of these patients presented any evidence of mental retardation or had first-degree family members with epilepsy or febrile seizure history. Eight patients with well characterized antecedent prolonged febrile seizures that occurred within 6 months and 4 yrs of age were classified as FS cases. The average and standard error for disease onset age and IPI in this subgroup were 7±1.8 yrs. and 2±0.5 yrs. respectively. The remaining patients had no history of febrile IPI (NFS cases) and their average and standard error for t disease onset age and IPI were 11.9±1.8 yrs. and 8.8±2.2 respectively. Patient's age at surgery ranged from 13 to 57 years (mean age 32 years). Average duration of epilepsy before surgery was 21.9±6 years for FS and 22.1±2.2 years for NFS cases. Genomic studies of hippocampal CA3 encompassed 6 FS and 12 NFS cases. MRI studies included 4 FS and 8 NFS cases.

**Table 1:** Patients' clinical and pathological data.

				Epilepsy			Age at	
Patient			Febrile	IPI	onset	duration	surgery	
ID	Gender	FR^c^	seizure	(yr/mo)	(yr)	(yr)	(yr)	Side
FS1^a^	M	no	yes	4	4	9	13	right
FS2^a^	M	no	yes	1/8	7	18	25	left
FS3^b^	F	no	yes	1	8	11	19	right
FS4^a^	M	no	yes	4	4	26	30	right
FS5^b^	M	3^rd^	yes	0/6	15	14	29	left
FS6^a^	F	no	yes	2	14	5	19	right
FS7^a,b^	M	2^nd^	yes	2	2	37	39	left
FS8^a,b^	F	no	yes	0/6	2	55	57	right
NFS9^a^	F	no	no	7	7	21	28	left
NFS10^a^	M	no	no	1/6^d^	5	27	32	left
NFS11^a^	F	no	no	4^d^	4	19	23	left
NFS12^a^	F	no	no	13	13	29	42	left
NFS13^b^	M	no	no	0/9^d^	8	20	28	left
NFS14^b^	F	2^nd^	no	7	7	23	30	right
NFS15^a,b^	M	3^rd^	no	3	14	17	31	left
NFS16^a,b^	F	2^nd^	no	29	29	15	44	left
NFS17^a,b^	F	2^nd^	no	2	11	28	39	left
NFS18^a^	M	no	no	1	12	29	41	left
NFS19^a,b^	M	no	no	8	8	21	29	right
NFS20^a,b^	M	no	no	3	7	23	30	right
NFS21^b^	M	2^nd^	no	21^d^	23	10	33	right
NFS22^a^	F	2^nd^	no	18	18	7	25	left
NFS23^a,b^	F	3^rd^	no	13	13	42	55	right

**FR,** familial recurrence; ^a^case included in genomic analysis; ^b^case included in MRI studies; ^c^second or ^d^third degree relative with epilepsy; ^e^head trauma.

### Histopathology

**Figure 2:**
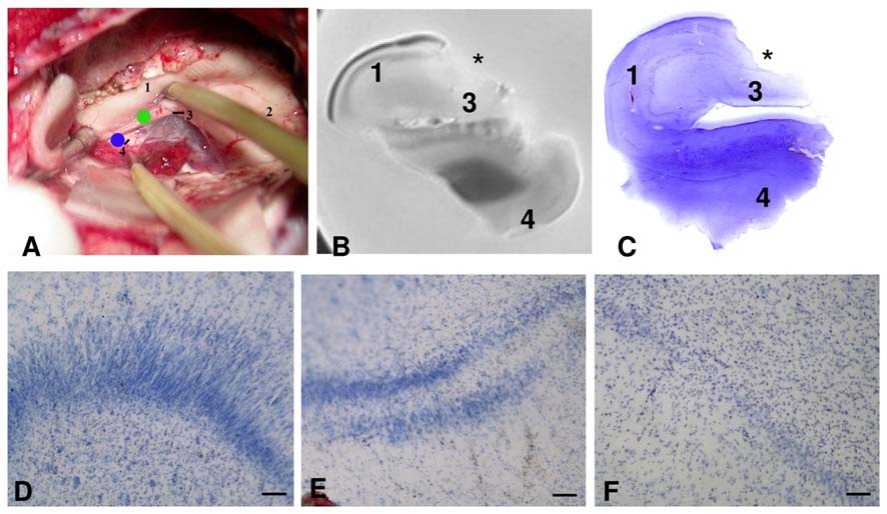
Tissue sampling and histopathological results. In **A,** surgical view of the hippocampus: (1) body of the hippocampus/CA1; (2) head of the hippocampus; (3) dentate gyrus/*fimbria* (green dot); (4) parahippocampal gyrus (blue dot). **B,** MRI of surgically resected hippocampus; **C**– **F** Nissl-stained hippocampal slices. In **B** and **C** the location of tissue resection for genomic analysis is marked with an asterisk showing the resection in the CA3–CA4 transition. **D**–**F** Cytoarchitectural alterations of the granule cell layer in sclerotic hippocampi. D: cell dispersion. E: bilamination. F: cell loss. Calibration bar  = 100 microns.

It was possible to confirm in all cases that tissue sampling for genomic analysis corresponded more precisely to the CA3–CA4 transition (Figure 2 A–C). Typical aspects of hippocampal sclerosis were observed for all cases, including gliosis and neuronal cell loss in CA1, CA3 and hilus of the dentate gyrus (CA4), with a relative preservation of CA2 and subicullum. In the granule cell layer (Figure 2 D–F) neuronal cell loss was significantly higher (p<0.05) in FS cases (Figure S1, supporting information), whereas no significant differences between FS and NFS were found for cell dispersion and bilamination.

### MRI texture analysis

A computational pipeline combining MRI high resolution acquisitions, image processing, texture analysis and pattern classification algorithms was used for image-based identification of histological features in the sclerotic hippocampi of 4 FS and 8 NFS cases. Classification procedures focused: i) cell loss and dispersion; ii) comparison between specimens from patients with or without antecedent febrile seizures history. The overall accuracies for correctly classified pixels were 90% for cell loss, 84% for cell dispersion and 90% for correlation with febrile history, thus showing that MRI texture features correlate with IPI.

### Transcriptome profile analysis

The comparison of CA3 global gene expression data between FS and NFS subgroups was performed using SAM procedure (5% false discovery rate) and yielded a total of 511 differentially expressed transcripts, all up-regulated (fold ≥3.0) in the FS subgroup. A subset of 335 genes annotated with Gene Ontology (GO) categories were found among these transcripts. Hierarchical clustering, performed by means of the TMEV 4.6.1 program, is presented as supporting information (Figure S2).

### Transcriptional interaction analysis

**Figure 3:**
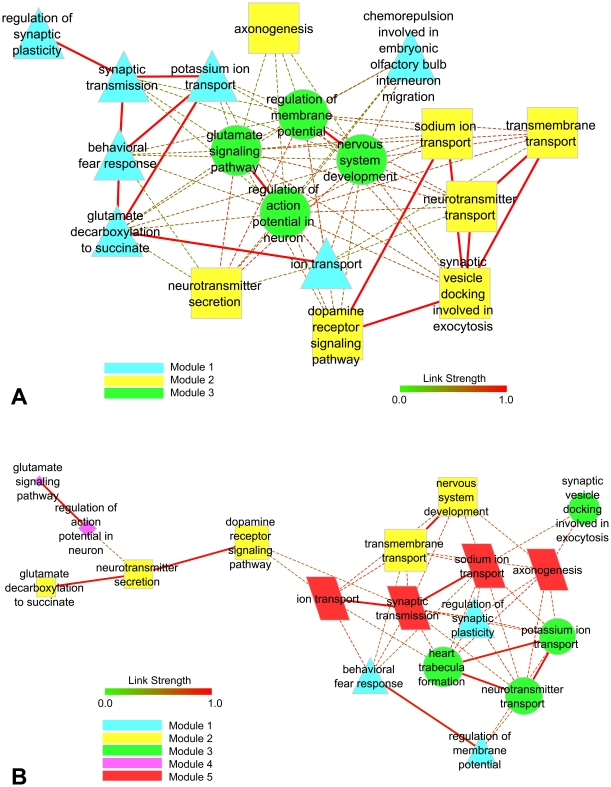
Theme proximity network analysis for GO biological processes categories. Thematic maps for FS and NFS subgroups appear in **A** and **B** respectively.

This analysis was accomplished using SAM-selected up-regulated GO annotated genes and the FunNet software. The strength of the links between each pair of genes was given by Spearman's correlation coefficient for expression profiles. The relevant biological themes, annotating differentially expressed genes, were indicated by significantly overrepresented categories from the GO Biological Processes (Figure 3). The overall distributions of the annotated genes by each GO category for FS and NFS subgroups appear as supporting information in Tables S1 and S2.

Figures 3A and 3B show the GO Biological Processes theme proximity networks for FS and NFS subgroups in that order. Intra- and inter-module links are indicated by solid or dotted lines, respectively. Although both networks comprised in essence the same themes, the sequence and link strengths of theme interactions were rather different. FS theme network was formed around three distinct modules: module 1 (triangle) displayed a theme cluster centered in synaptic transmission and ion transport; module 2 (square) was mainly related to dopamine signaling pathway, sodium ion transport and transmembrane transport; and module 3 (circle) was linked to glutamate signaling pathway and regulation of neuronal excitability. In the NFS network five distinct modules were formed (Figure 3B): modules 1 and 3 are mostly linked to synaptic plasticity and neurotransmitter transport, respectively; modules 2 and 4 are related to nervous system development and glutamate signaling pathway; and module 5 contains biological themes related to axonogenesis, ion transport and synaptic transmission.

**Figure 4:**
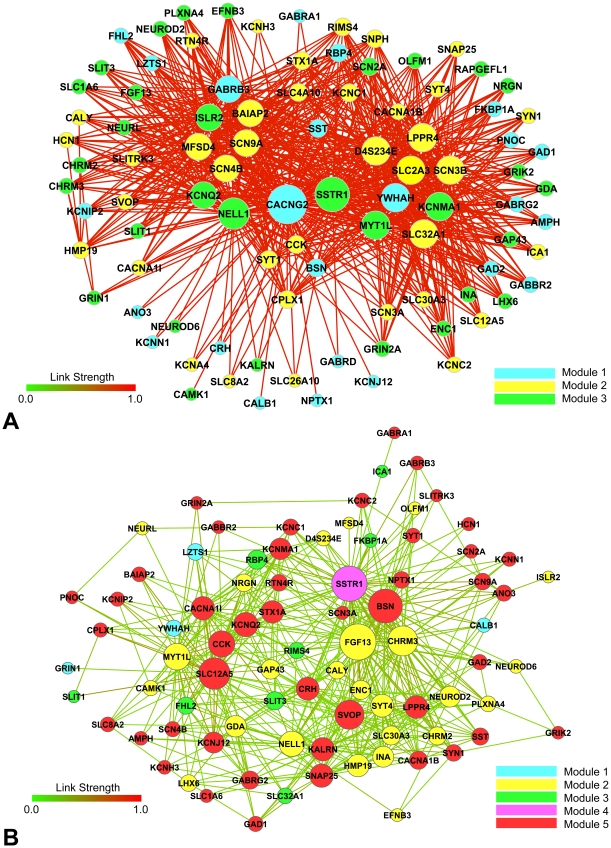
Transcriptional interaction analysis for GO molecular processes. Transcriptional interaction networks for FS and NFS subgroups appear in **A** and **B** respectively.

**Figure 5:**
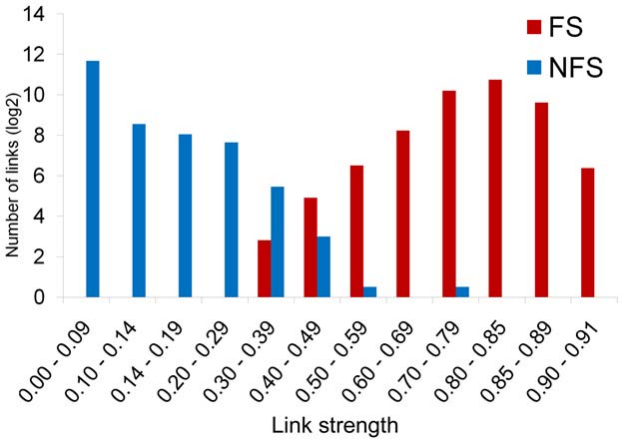
Gene-gene link strength category distribution. Number of gene-gene links distributed by link strength categories for FS (red bar) and NFS (blue bar).

The transcriptional interaction networks, or co-expression networks, for FS and NFS subgroups, depicted in Figures 4A and 4B respectively, were based on significantly over-represented GO Biological Processes categories. This analysis was accomplished using the Spearman's co-expression correlation for differentially expressed genes; therefore both networks were formed by the same set of differentially expressed genes. Co-expression coefficients ≥0.85, corresponding to 870 out of 4,186 links between each pair of genes, were selected for FS network. The NFS network was constructed adopting co-expression coefficients from 0.14 up to 0.70, corresponding to 518 out 4,186 links. In these networks the most relevant genes, or hubs, are the ones with the highest number of gene-gene links, graphically represented by the proportionally larger nodes. The number of gene-gene links distributed by link strength categories for FS and NFS subgroups is shown in Fig. 5. A list of the most relevant hubs according to the number of gene-gene links appears in Table 2, together with their respective biological functions.

**Table 2:** Main hubs in FS and/or NFS transcriptional interaction networks.

	Number of			
Gene	gene-gene links		Gene product and/or biological function	Refs^b^
	FS	NFS		
CACNG2	**91**	none^a^	stargazin;	*24*
			transmembrane AMPA receptor regulatory protein	
SSTR1	**76**	**50**	somatostatin receptor 1	*27*
NELL1	**65**	**29**	neural differentiation control	*29*
SLC32A1	**55**	9	GABA vesicular transporter	*32*
MYT1L	**55**	**32**	regulation of neurotranscription & neural differentiation	*40*
KCNMA1	**55**	21	calcium-ativated potassium channel	*30*
YWHAH	**55**	10	mediation of signal transduction in neurons;	*37*
			synaptic transmission	
SLC2A3	**55**	None^a^	alias GLUT3; main neuronal glucose transporter	*36*
SCN3B	**55**	None^a^	sodium channel beta 3 subunit; sodium ion transport	*31*
D4S234E	**55**	8	alias NEEP21; AMPA receptor recycling in neurons	*34*
LPPR4	**55**	**24**	(alias PRG1)	*35*
			control of hippocampal excitatory transmission	
KCNQ2	**49**	**27**	voltage-gated potassium channel	*41*
SCN4B	**49**	8	voltage-gated sodium channel type IV, beta	*42*
SCN9A	**49**	11	voltage-gated sodium channel type IX	*43*
MFSD4	**49**	1	major facilitator superfamily containing domain 4,	
			involved in nerve damage repair	*47*
ISLR2	**49**	1	modulation of growth factors signals during	*46*
			neural development	
BAIAP2	**49**	7	regulation of dendritic spine morphogenesis and density	*45*
GABRB3	**49**	5	GABA(A) receptor (GABAR) beta 3 subunit	*44*
FGF13	10	**52**	(alias FHF2) biding/modulation of voltage-gated sodium	*48,49*
			channel;nervous system development	
CHRM3	11	**39**	cholinergic neuromodulation of hippocampal circuitry	*52*
BSN	18	**47**	(Bassoon) speeding of vesicle reloading at	*51*
			excitatory synapses	
SLC12A5	10	**42**	(alias KCC2) potassium-chloride cotransporter;	*54*
			modulation of neuronal excitability	
CCK	18	**31**	output regulation of distinct types of inhibitory	*57*
			interneurons in the hippocampus	
SVOP	18	**37**	transporter-like protein that localize to	*77*
			neurotransmitter-containing vesicles	
KARLN	3	**25**	Kalirin, brain-specific guanine-nucleotide exchange	*59*
			factor (GEF) for the Rho family of small GTPases;	
			maintenance of hippocampal pyramidal neuron dendrites	
			and dendritic spines	
SNAP25	10	**25**	SNARE superfamily protein; regulation of synaptic	*60*
			vesicle exocytosis	
CACNA1I	10	**25**	Voltage-dependent calcium channel controlling	*58*
			rapid entry of Ca(2+)	

a Link strength coeficient <0.10. ^b^References. Bold numbers indicate hubs in FS and/or NFS subgroups.

### FS co-expression network

The gene CACNG2 formed the major hub (91 gene-gene links) and it was exclusive of this network. This gene codes for stargazin, a transmembrane receptor regulatory protein involved in the control of surface and synaptic expression of the excitatory AMPA-type glutamate receptors [24], [25]. The gene SSTR1 was the second FS major hub (76 gene-gene links) and, interestingly, it was also the major hub of the NFS network (see below). This gene is very relevant for epilepsy since it codes for a receptor of the endogenous antiepileptic somatostatin [26], [27]. The third FS major hub (65 gene-gene links) was the gene NELL1 (neural epidermal growth factor-like 1, [28], which codes for a protein firstly found in neuroblastoma cell lines and possibly involved in controlling neural differentiation [29].

Two significant secondary hub clusters were identified in the FS network. One of these clusters, in which all hubs had 55 gene-gene links, comprised: i) the genes encoding the voltage-gated potassium (KCNMA1) and sodium (SCN3B) channels [30], [31]; ii) SLC32A1, a gene coding for a vesicular GABA transporter [32]; iii) D4S234E, alias NEEP21, which codes for a protein with a pivotal role in the hippocampal recycling and internalization of AMPA-receptors [33], [34]; iv) LPPR4, a gene involved in the modulatory control of hippocampal excitability, preventing hyperexcitability in glutamaergic neurons [35]; v) SLC2A3, alias GLUT3, the main neuronal glucose-transporter [36]; vi) YWHAH, a candidate gene for autosomal dominant familial partial epilepsy with variable foci [37], which codes for a neural protein that mediates signal transduction by binding to phosphoserine-containing proteins [38], [39] and vii) MYT1L, a zinc finger gene involved in the recruitment of histone deacetylase to regulate neural transcription [40].

The other cluster, with 49 gene-gene links per hub, included genes codifying for voltage-gated potassium (KCNQ2, [41]) and sodium (SCN4B, [42]; SCN9A, [43]) channels and for the GABA_A_ receptor subunit (GABRB3, [44]), plus the genes BAIAP2, which codes for an adaptor protein highly expressed at postsynaptic density of excitatory synapses, regulating dendritic spine morphogenesis and density [45], ISLR2, involved in the modulation of growth factor signals during neural development [46] and MFSD4, which codes for a protein (major facilitator superfamily domain containing 4) that interacts with ELK transcription factors (up-regulated by nerve damage) in several regions of the nervous system [47].

### NFS co-expression network

Here the gene SSTR1 appeared again as a major hub (50 links), but together with FGF13 (52 links), which codes for a voltage-gated sodium channel modulator [48] and is involved in nervous system development [49], BSN (47 links), that codes for Bassoon, a protein engaged in organizing Ca^2+^ channel clustering, synaptic vesicle docking and speeding of synapse reloading at excitatory synapses [50], [51] and CHRM3 (39 links), a muscarinic acetylcholine receptor involved in the cholinergic neuromodulation of hippocampal circuitry [52].

Two relevant secondary hub clusters, predominantly related to NFS thematic modules 2 (nervous system development) and 5 (axonogenesis, ion transport), were identified in this network. One was formed around the gene SLC12A5 (42 links), alias KCC2, a gene encoding the neuron-specific K-Cl cotransporter involved in the modulation of GABA-ergic transmission [53] and with a central role in promoting inhibition and preventing hyperexcitability in human hippocampus [54], [55]. The other was centered in SVOP (37 links), a gene encoding for a transporter-like protein localized to neurotransmitter-containing vesicles [56]. The SLC12A5 cluster included MTY1L, the axonogenesis-related gene CCK [57], KCNQ2 and CACNA1I, which codes for a T-type calcium channel [58]. The SVOP cluster contained as significant hubs the genes NELL1 and KARLN [59], both related to neural development, and SNAP25, a member of the SNARE superfamily involved in the regulation of synaptic vesicle exocytosis [60]. Additionally, overall gene-gene link strength was diminished in relation to the values observed for the FS network (see Figure. 5), possibly reflecting higher phenotypic and clinical heterogeneities among afebrile cases. The significance of these findings for clarifying the molecular pathomechanisms involved in RMTLE and for the identification of novel and/or phenotype-specific therapeutic target candidates will be discussed below.

### qPCR Validation

In order to validate the DNA microarray data five up-regulated genes - NELL1, NEURL, NEUROD6, SVOP and SYT1 - were selected for real-time quantitative PCR (qPCR) analysis. The fold-changes for each gene, comparing FS versus NFS group's average relative gene expression, confirmed DNA microarray results (Figure S3).

## Discussion

Through the use of a systems biology approach, based on the comparative analysis of CA3 transcriptional interaction networks from RMTLE patients with (FS) or without (NFS) antecedent childhood febrile seizures, we were able to demonstrate that febrile and afebrile RMTLE constitute two distinct genomic phenotypes. MRI texture analysis of dentate gyrus also differentiated FS and NFS subgroups and histological examination showed higher neuronal cell loss in FS hippocampi, thus representing a morphological counterpart for the genomic findings. The uniqueness of FS genomic signature is supported by a recent epidemiologic survey on temporal lobe epilepsy (TLE) patients with (TLE-FS) or without (TLE-NFS) early childhood febrile seizures based on age of onset, seizure type and ictal symptoms and familial recurrence, that placed TLE-FS as phenotype distinct from afebrile TLE [61]. Similar conclusions came from a study of neuropathologic features of resected hippocampi from RMTLE patients where morphological alterations such as cell genesis and granule cell dispersion correlate with febrile IPI [62]. Therefore, the above-mentioned genomic and clinicopathological evidences confirm early clinical, imaging and epidemiological observations [3], [10], [63]–[66] pointing out to the relevance of febrile IPI in RMTLE.

Co-expression network analyses of FS and NFS transcriptomes revealed relevant commonalities and differences. The most striking commonality involves the gene SSTR1, a major hub in both networks. The SSTR gene family codes for the brain receptors of somatostatin (SST), a potent endogenous antiepileptic [26]. In rodents SST acts on dentate gyrus, CA1 and CA3 reducing epileptiform events. Therefore SST receptors expressed in these hippocampal areas are considered potential therapeutic targets for treating refractory temporal lobe epilepsy [27]. In rodents receptor subtypes 2 and 4 appear to mediate the majority of the antiepileptic actions of somatostatin [27]. In humans there are five SSTR genes [67] and SSTR2 was found to be downregulated in CA1 and CA3 hippocampal explants surgically obtained from patients with temporal lobe epilepsy, probably reflecting increased neural cell loss in these areas and also the SSTR2 involvement in antiepileptic processes [68]. Here we show that SSTR1, which is known to be expressed in human fetal central nervous system [69], is also expressed in CA3 hippocampal region of patients with RMTLE. Thus SSTR1 and its product could be thus considered as potential therapeutic targets in RMTLE.

Two genes related to nervous system development, MYT1L and NELL1, also appeared as significant hubs in both networks. MYT1L is a neural specific zinc finger gene regulating neuronal transcription [40], [70], oligodendrocyte differentiation [71] and cortical neuron progenitor development [72]. NELL1 codes for a protein containing EGF-like repeats and its expression has been detected in fetal brain [28] and in central nervous system tumors [29], being probably active in controlling neural differentiation pathways. To our knowledge, this is the first description of the expression of both genes in human CA3 hippocampal cells. This expression could interpreted as compensatory to the decreased hippocampal neurogenesis observed in temporal lobe epilepsy [18], [19], which is particular severe in dentate gyrus and CA1 and CA3 in RMTLE [62]. Several strategies have been devised to augment neurogenesis in the dentate gyrus in temporal lobe epilepsy [19]. However, it seems unsafe to consider MYT1L and NELL1 in the scenario of gene therapy: the first has a widespread role in controlling neural differentiation processes through gene silencing [40] and the former is related to neoplastic processes in the brain. Moreover, there always the risk of promoting erroneous incorporation of newly formed neurons in hippocampal circuitry [19]. The same rationale discussed above applies to the nervous system development-related hubs ISLR2 and BAIAP2 in the FS network, as well as to the NFS axonogenesis-related secondary hubs CCK and KARLN.

The significant hub differences among FS and NFS interaction networks encompass genes that fall in two main categories: modulators of neurotransmission and modulators of intrinsic membrane excitability. The modulators of neurotransmission include genes related to i) glutamatergic pathways and excitatory transmission; ii) GABAergic transmission and iii) synaptic transmission. These hub differences and their biological significance are briefly discussed below.

The genes acting on glutamatergic pathways and excitatory transmission have more relevance (considering gene-gene links) in the FS network, forming two of its major and exclusive hubs, namely CACNG2 (stargazin) and SLC2A3 (alias GLUT3). The first is essential for stabilizing diffuse AMPA receptors in the post synaptic density, an important feature of glutamatergic synaptic transmission [73]. The second is the main neuronal glucose transporter and modulates brain glucose uptake, being therefore relevant for excitatory transmission processes [74]. The genes D4S234 (alias NEEP21), involved in AMPA receptor recycling in neurons [33] and LPPR4 (alias PRG1), a controller of hippocampal excitatory transmission [35] also constitute major FS hubs related to neurotransmission modulation (Table 2), whereas BSN, a gene engaged in the speeding of vesicle reloading at excitatory synapses [50] is the sole major NFS hub in this group, with LPPR4 constituting a secondary hub.

Regarding the modulators of GABAergic transmission, the major FS hubs are GABRB3, a gene coding a GABA_A_ receptor subunit and in which mutations are known to cause childhood absence epilepsy [44], and SLC32A1, that codes for a vesicular GABA transporter [32]. In the NFS network the epilepsy-associated gene SLC12A5 (alias KCC2) appears as a very relevant hub. KCC2 by governing cellular chloride concentrations enables GABA-A receptor activation to hyperpolarize the cell and may represent an intrinsic antiepileptogenic mechanism [75] Loss of KCC2 may render activation of the receptor depolarizing. Reduction of its expression in subicular regions adjacent to sclerotic areas of the hippocampus has been associated to epileptiform activity [55].

The four genes related to neurotransmission modulation form relevant hubs only in the NFS co-expression network. Three of these genes are involved with synaptic vesicle function: SVOP, a nucleotide and ion transporter-protein [76], [77]; SNAP-25, a fundamental component of the SNARE complex used for fast synaptic communication in excitatory and inhibitory circuits [60] and BSN, that codes for Bassoon, a protein engaged in the speeding of synapse reloading at excitatory synapses [50], [51]. The fourth gene in this group is CHRM3, is a muscarinic acetylcholine receptor that confers differential cholinergic modulation to neurochemically distinct hippocampal basket cell subtypes [52]. Interestingly, early life seizures appear to increase the efficacy of muscarinic receptors coupling to protein G accounting for adult susceptibility to epilepsy, what makes this gene a potential target for novel anticonvulsant drugs [78].

In the category of modulators of intrinsic membrane excitability**,** the FS network has as significant hubs KCNMA1, a gene involved in the regulation of action potential in neurons and whose defective expression plays a critical role in the pathogenesis of mesial temporal lobe epilepsy [30] and SCN3B, which codes for a calcium-activated potassium channel and whose downregulation is associated to hippocampal sclerosis [23], [31]. Interestingly, this gene has 55 gene-gene links in the FS network but none in the NFS (Table 2). Also in the FS network are the genes SCN4B/ SCN9A, involved in sodium ion transport, and KCNQ2, a voltage-gated potassium channel, all relevant for the regulation of neuronal excitability. The NFS network displays in this category only two significant hubs formed by the genes FGF13, a voltage-gated calcium channel modulator, and CACNA1I, a calcium-signaling gene responsible for neuronal excitation [79].

The general picture that emerges from the analysis of co-expression networks shows that the FS distinctive hubs are mostly linked to glutamatergic signalization while in the NFS network the majority of the distinctive hubs involve GABAergic pathways and neurotransmission modulation. Both networks have relevant hubs related to nervous system development, what is consistent with the cell genesis activity described in the hippocampus of RMTLE patients [19], [62]. Moreover, two candidate genes for therapeutic targeting came out from this analysis: SSTR1, a relevant common hub in febrile and afebrile transcriptomes, and CHRM3, due to its putative role in epilepsy susceptibility development.

In conclusion, the experimental design of this work, centered on the comparative studies of CA3 transcriptome and of dentate gyrus morphological features, was instrumental for characterizing febrile RMTLE as a distinct phenotype. The co-expression network analysis permitted to reveal molecular mechanisms underlying the refractory epilepsy phenotypes, pointing out two potential therapeutic targets represented by the genes SSTR1 and CHRM3. Furthermore, the biological system approach adopted here proved to be a powerful tool for exploring the intricacies of the epilepsy molecular mechanisms, allowing the functional profiling of a large set of genomic data.

## Methods

### Patients

The RMTLE patients included in this study were selected through the CInAPCe-FAPESP Program (www.fapesp.br/en/; www.cinapce.org.br), the largest Brazilian research program on epilepsy and neuroimaging. This research has been approved by the research ethics committees of Hospital das Clínicas da FMUSP and of Hospital Albert Einstein under numbers 251/05 and CAEE 0122.0.028.174.05 respectively. A written informed consent was obtained from all patients. Refractory epilepsy cases were defined as those who have not gained seizure control after treatment with three or more anticonvulsant drugs. All patients were submitted to clinical, electrophysiological, neuropsychological and neuroimaging evaluations before surgery. The patients were submitted to epilepsy surgery between February 2009 and August 2010.

### Brain tissue specimens for gene expression and neuropathological studies

Fresh ex-vivo explants from hippocampal CA3 of our patients were obtained at the surgery room (Figure 2A) and immediately preserved with RNAlater (Qiagen cat. no. 76106, Valencia, CA). The entire hippocampus was then removed. MRI and histological studies were accomplished in all removed hippocampi for neuropathology analysis and for confirming that the explants for gene expression were obtained at the proper site (Figure 2B–C).

### RNA extraction

Brain tissue explants from CA3 (3–4 mm^3^) were homogenized with TissueRupter (Qiagen, cat. no. 9001272 Valencia, CA) and total RNA was extracted from the homogenates using the RNeasy Lipid Tissue Kit (Qiagen cat. no. 74804, Valencia, CA) according to the manufacturer's instructions. RNA quality was assessed on the Agilent BioAnalyzer 2100 (Agilent, Santa Clara, CA). All samples were stored at −80°C until used in hybridization experiments.

### Microarray hybridization and gene expression analysis

In order to determine gene expression profiles, 44 K DNA microarrrays (Agilent Technologies, cat no. G4845A Santa Clara, CA) were used. The procedures for hybridization followed the protocols provided by the manufacturer's instructions (One-Color Microarray-Based Gene Expression Analysis - Quick Amp Labeling). The images were captured by the reader Agilent Bundle according to the parameters recommended for bioarrays and extracted by Agilent Feature Extraction software version 9.5.3. Among the 45,015 spots present in each array only those with none or only one flag (i.e. low intensity, saturation, controls, etc.) were selected for analysis using the R software version 2.11.1 (R Development Core Team, 2010) and the Lowess test for normalization. We identified 28,546 valid transcripts for the CA3 samples (6 FS and 12 NFS patients). By means of the TMEV software version 4.6.1 [80] we selected differentially expressed transcripts using the Significance Analysis of Microarrays (SAM) procedure, a statistical technique for finding significant genes in a set of microarray experiments [81]. The input data is gene expression measurements from a set of microarray experiments, as well as a response variable from each experiment. It uses repeated permutations of the data to determine if the expressions of any genes are significantly related to the response. The cutoff for significance is determined by a tuning parameter delta, chosen by the user based on the false discovery rate. Here we used a 5% false discovery rate.

Hierarchical clustering was based on Pearson correlation and complete linkage. All microarray raw data has been deposited in GEO public database (http://www.ncbi.nlm.nih.gov/geo), a MIAME compliant database, under accession number GSE28674.

### Transcriptional interaction analyses (GO and Network analysis)

We used the FunNet software (http://www.funnet.info) - based on the Gene Ontology Consortium (GO, http://www.geneontology.org) genomic annotations - for performing the functional profiling of gene expression data and identifying the biological themes and gene-gene interactions in which the differentially expressed genes are involved. The parameters used in these analyses were Spearman co-expression correlation and 5% false discovery rate. Themes with significant relationship in the transcriptional expression space were associated to build transcriptional modules in a proximity network. A transcriptional interaction network, corresponding to the theme proximity network, was then obtained. Data analysis and visualization were accomplished through Cytoscape (version 2.8.0, www.cytoscape.org).

### qPCR

Differential gene expression data were validated through quantitative real-time polymerase chain reaction (qPCR). Specific primers for five selected genes (Table S3) were designed using the Primer-BLAST (Primer3 Input, version 0.4.0 and BLAST, available at http://www.ncbi.nlm.nih.gov/tools/primer-blast/). All samples were amplified in triplicates. Amplification reactions were performed in a 25 uL final volume containing 1X SYBR Green mix (*Quantitec SYBR Green PCR kit*, QIAGEN, Hilden, DE), 10 pmol of each *primer* and 2 µL cDNA (1/10 dilution, synthesized from 1 µg of total RNA). Real time PCR amplifications were performed in Applied Biosystems StepOne Plus Real Time PCR System with StepOne software (Applied Biosystems, Forrest City, CA, USA) with the following cycling parameters: an initial hot start of 95°C for 15 min followed by 50 cycles of 95°C for 15 s and 60°C for 30 s. In order to normalize qPCR reactions, *GAPDH* was included as reference gene after checking that amplification curves for different 12 different CA3 RNA samples (6 from FS and 6 from the NFS patients) yielded essentially the same results. Relative expression was determined by the relative standard curve method and presented as fold change comparing FS versus NFS mean values.

### Neuropathology

Specimens obtained during epilepsy surgery were fixed in 4% paraformaldehyde in 0.1 M phosphate buffer for 24 h and then transferred to a solution of PF 1% plus sucrose 30% in PB 0.1 M for one week. Each hippocampus (25–30 mm length) was carefully oriented, trimmed and sectioned in the plane perpendicular to its longitudinal axis. The entire hippocampus (head and body) was used in the present investigation (50–60 slices/patient). Because sections cut tangentially to the principal cell layers or at inconsistent angles from the longitudinal axis of the hippocampus can produce unusual histological features, considerable care was taken to ensure that all hippocampal sections were cut in a plane strictly perpendicular to the longitudinal axis of the hippocampus. Sixty-micron coronal slices through the entire extension of the hippocampus were obtained using a cryostat (−21°C). One out five slices were mounted on glass slides and stained with cresyl violet (Nissl). The histological pattern described in the [82] atlas was used to identify the normal cytoarchitectural features in every hippocampal section.

Semi-quantitative assessment of sclerotic hippocampi was made for each histological slice focusing on dentate gyrus abnormalities at hippocampal body. The following parameters were evaluated: gliosis, neuronal cell loss and cytoarchitectural disorganization (dispersion and bilamination). These features were graded from zero (no abnormality) to 3 (very intense abnormality). An additional grade 4 was included for neuronal cell loss, indicating absence of neurons in the region under examination. The mean value for each parameter was calculated in each individual case. Bilamination of granular cell layer was described as present (yes) or absent (no). Statistical analysis was accomplished using non-parametric Mann-Whitney t-test, p<0.05.

### Dentate Gyrus MRI texture analysis

Image acquisitions of 12 hippocampi surgically obtained from 4 FS and 8 NFS cases (see Table 1) were performed in a 3.0T SIEMENS Magnetom TIM Trio scanner (80 cm bore, 40 mT/m, 230 mT/m/s) using a surface coil (“Loop 7”). Images were acquired with the hippocampus immersed in 1% paraformaldehyde in 0.1 M phosphate buffer, inside a BD Falcon™ 50 cc conical tube. High-resolution images were acquired using a TSE (Turbo Spin Echo) protocol, with TR = 3700 ms, TE = 76 ms, fat sat by IR, TF = 7, FA = 180, BW = 40, FOV = 43 mm (70% AP phase oversampling), a 512×512 matrix, slice thickness of 1.6 mm and 32 NEX. Attained in-plane resolution is 80 µm×80 µm and CNR = 15. In order to guide data extraction, regions of interest covering the dentate gyrus were manually drawn over the MRI images by a neuropathologist. Imaging data was then processed in a pipeline [83] consisting of preprocessing (noise filtering, background segmentation, intensity normalization), feature extraction (texture calculation) and analysis (data randomization, data resampling, and classification). Feature extraction was performed with the aid of a 9×9 points sliding window using a set of 150 texture parameters calculated for every pixel in the image, namely: co-occurrence matrix parameters [84]; run-length matrix parameters [85]; wavelet parameters [86]; fractal dimension [87]; Markov random field parameters [88] and Gabor filter parameters [89], [90].?A data matrix containing texture information from the dentate gyrus of FS and NFS patients was assembled using Matlab (http://www.mathworks.com/products/matlab/). Classification procedures were executed using a random forest algorithm and the Weka [91] data mining workbench. Since the number of NFS patients was larger, the matrix was processed so to keep the number of samples equal for both classes thus avoiding bias in the classification.?Results were attested using a 10-fold cross-validation methodology, where the entire data set is divided in 10 partitions and the process of training/testing is repeated 10 times. In each turn the classifier was calculated for 9 partitions and tested for the remaining one. The final results are the mean output of each turn. The kappa statistics was used to verify if the agreement between classification and true classes exceeds chance levels. Values higher than zero indicate that the classification results were not merely caused by chance. Finally, a Receiver Operator Characteristic (ROC) curve was used to assess the quality of the trained classifier. A detailed description of the use of this methodology for assessing hippocampal sclerosis in MTLE will appear in another paper of our group headed by MC Alegro.

## Supporting Information

Figure S1 **Dentate gyrus histological features.** Neuronal cell loss and cell dispersion values are shown for FS (gray) and NFS (white) tissue samples. Histological features were graded from zero (no abnormality) to 3 (very intense abnormality).
(TIF)

Figure S2 **Hierarchical clustering for differential expressed genes.** Pearson correlation hierarchical clustering of 335 differentially expressed annotated genes across the FS and NFS subgroups.
(TIFF)

Figure S3 **qPCR validation of DNA microarray data.** In **A** the boxplots comparing the DNA microarray expression values of five selected genes in FS (gray) and NFS (white) samples. In **B** qPCR expression fold changes comparing FS X NFS samples for the same genes showing upregulation in FS.
(TIF)

Table S1 Gene classification in GO Biological Process for FS group.
(DOC)

Table S2 Gene classification in GO Biological Process for NFS group.
(DOC)

Table S3 Primer sequences used for validation of gene expression by qPCR.
(DOC)
