# Supplementary figures and images for: Hippocampal CA3 Transcriptome Signature Correlates with Initial Precipitating Injury in Refractory Mesial Temporal Lobe Epilepsy
Source: PLoS One. 2011 Oct 14;6(10):e26268. doi: 10.1371/journal.pone.0026268 (PMC3194819; doi:10.1371/journal.pone.0026268)

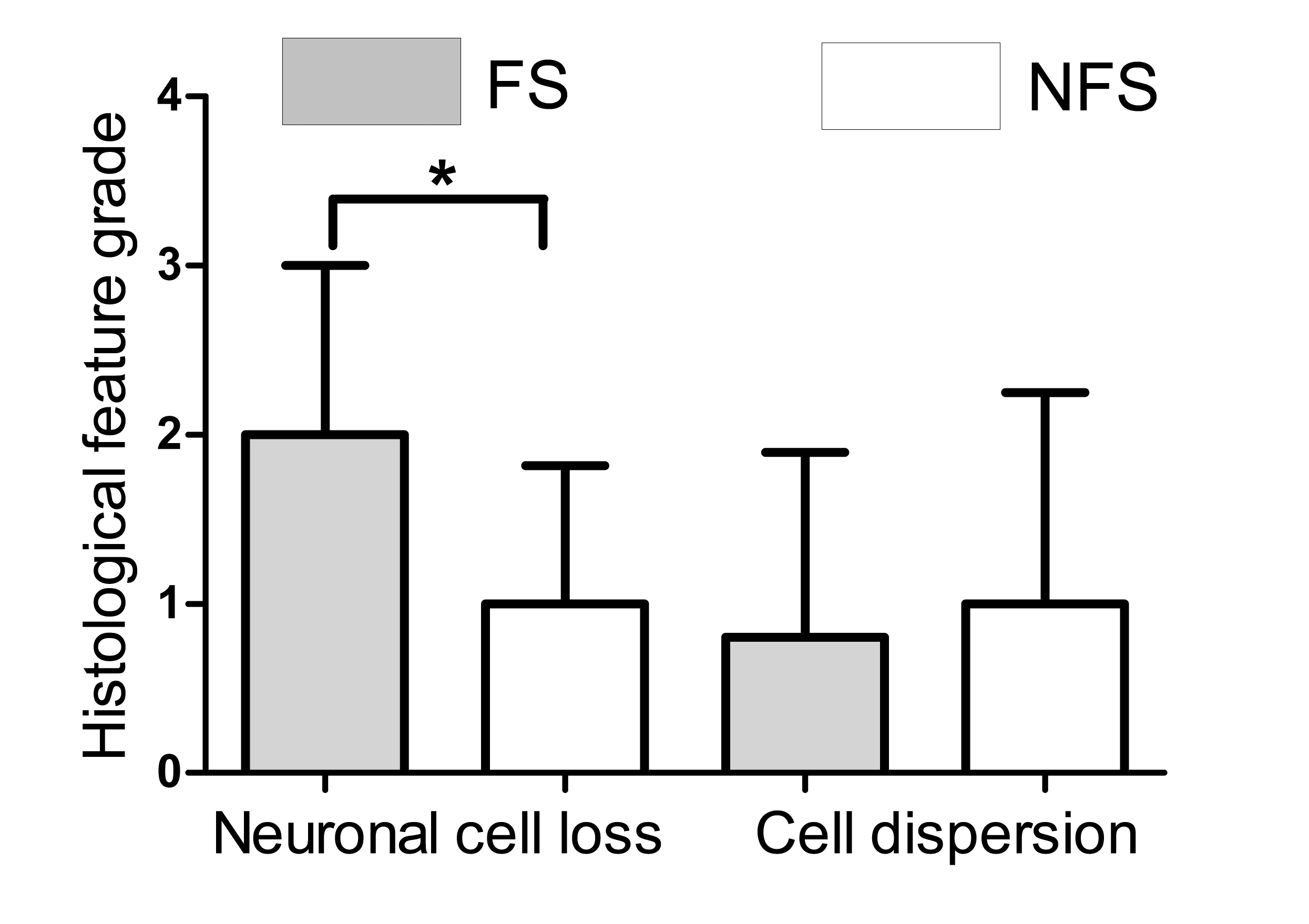

Supplement: Figure S1 — Dentate gyrus histological features. Neuronal cell loss and cell dispersion values are shown for FS (gray) and NFS (white) tissue samples. Histological features were graded from zero (no abnormality) to 3 (very intense abnormality). (TIF) [file pone.0026268.s001.tif]

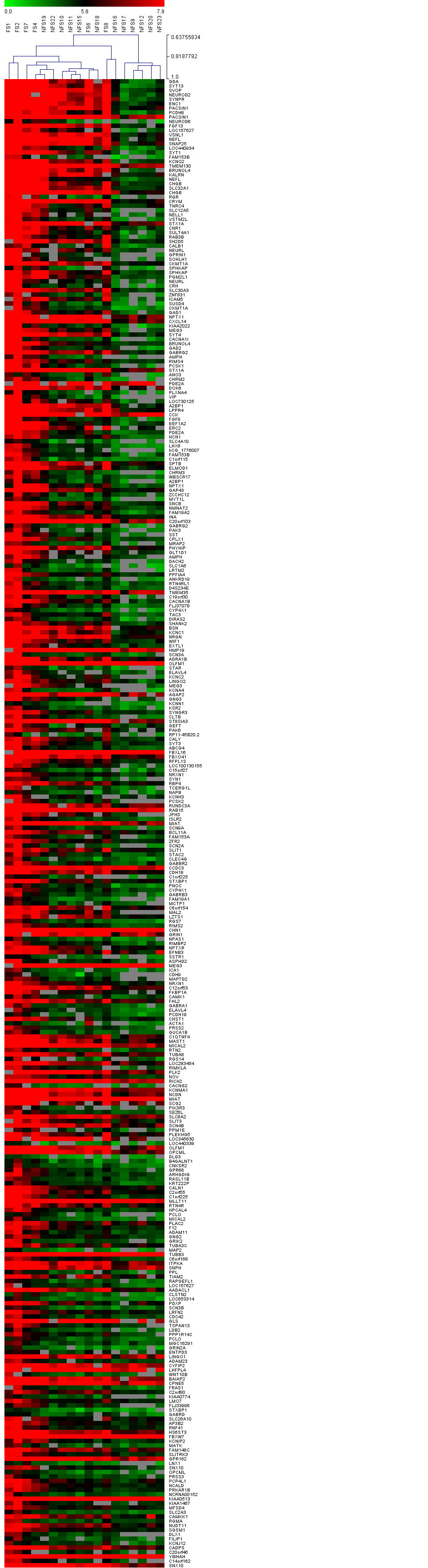

Supplement: Figure S2 — Hierarchical clustering for differential expressed genes. Pearson correlation hierarchical clustering of 335 differentially expressed annotated genes across the FS and NFS subgroups. (TIFF) [file pone.0026268.s002.tiff]

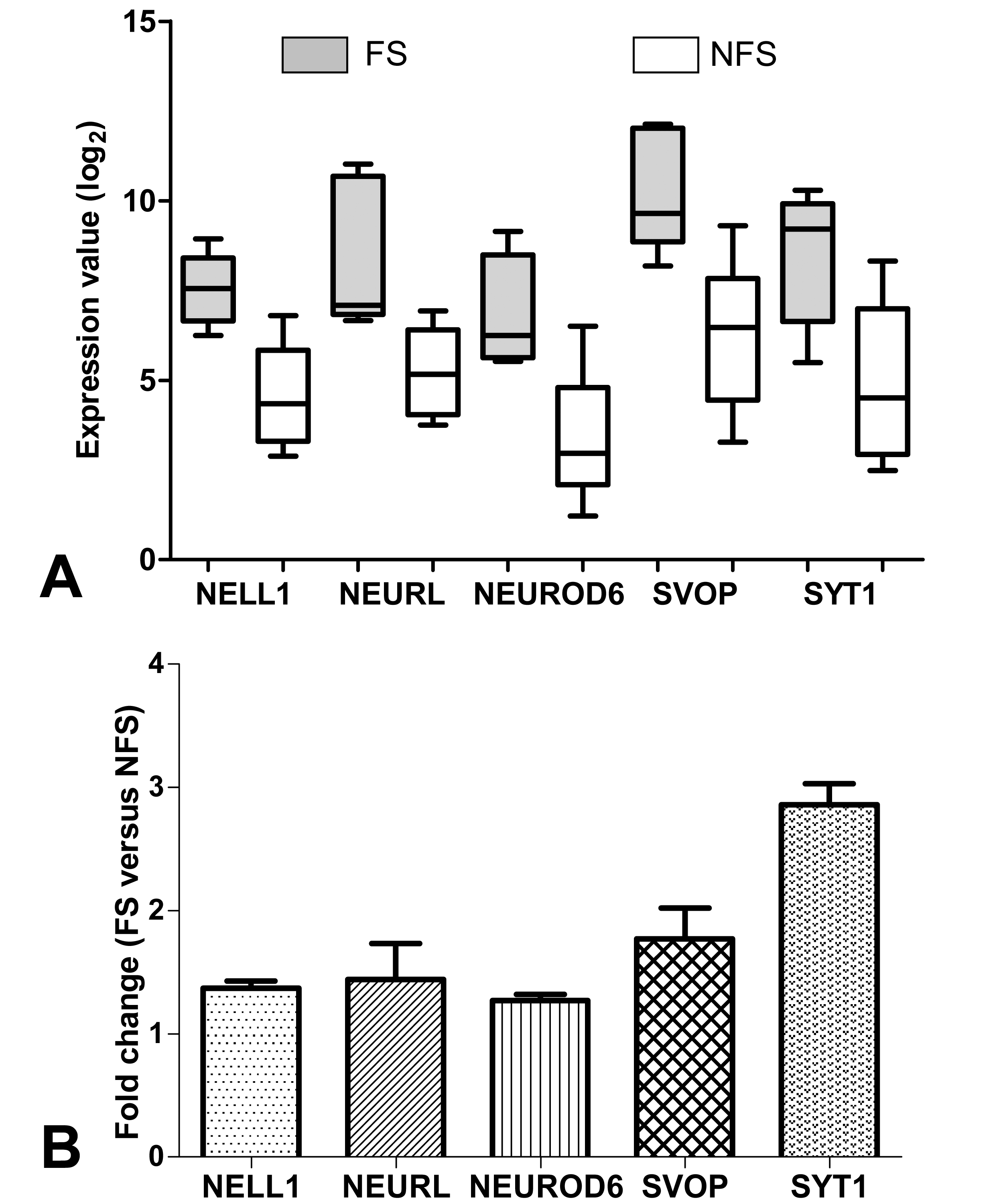

Supplement: Figure S3 — qPCR validation of DNA microarray data. In A the boxplots comparing the DNA microarray expression values of five selected genes in FS (gray) and NFS (white) samples. In B qPCR expression fold changes comparing FS X NFS samples for the same genes showing upregulation in FS. (TIF) [file pone.0026268.s003.tif]
