# Supplementary material for: Hippocampal CA3 Transcriptome Signature Correlates with Initial Precipitating Injury in Refractory Mesial Temporal Lobe Epilepsy
Source: PLoS One. 2011 Oct 14;6(10):e26268. doi: 10.1371/journal.pone.0026268 (PMC3194819; doi:10.1371/journal.pone.0026268)
Supplement: Table S1 — Gene classification in GO Biological Process for FS group. (DOC) [file pone.0026268.s004.doc]

**Table S1.** Gene classification in GO Biological Process for FS group.
