# Supplementary material for: Hippocampal CA3 Transcriptome Signature Correlates with Initial Precipitating Injury in Refractory Mesial Temporal Lobe Epilepsy
Source: PLoS One. 2011 Oct 14;6(10):e26268. doi: 10.1371/journal.pone.0026268 (PMC3194819; doi:10.1371/journal.pone.0026268)
Supplement: Table S3 — Primer sequences used for validation of gene expression by qPCR. (DOC) [file pone.0026268.s006.doc]

**Table S3**. Primer sequences used for validation of gene expression by qPCR.

| Gene name | Primer foward (5' - 3') | Primer reverse (5' - 3') | Product lenght (pb) |
| --- | --- | --- | --- |
| NELL1 | TGCCTGTTGTGACTTTTGTCCCA | TGGCTTCAGCCTGTTTAAGCT | 165 |
| NEURL | TGTGCTGGCCTTGCAGTGG | CCCAACCCTCAGCCTCTGG | 158 |
| NEUROD6 | GTATTTGCAGATGGGGCAAC | CCTTAAAAATGTGGGGTGGA | 174 |
| SVOP | CCGTGGAGTGGAGAGGGGT | CCCCTGCCTTCACCATGGGA | 160 |
| SYT1 | CACATGTACCCTTCTGACAAAG | TAGTTGGCTCGCAGGCCAC | 182 |
| GAPDH | ACCACAGTCCATGCCATCAC | TCCACCACCCTGTTGCTGTA | 452 |
